# Oxygen supplementation in ambulatory patients with heart failure: a randomized proof-of-concept study

**DOI:** 10.1093/ehjopen/oeaf074

**Published:** 2025-06-11

**Authors:** Maxime Tremblay-Gravel, Anna Nozza, Stanislav Glezer, Alan Kamada, Jacinthe Boulet, Marie-Claude Parent, Geneviève Giraldeau, Normand Racine, Anil Nigam, Isabelle Cloutier, Raynold Pierre, Jean-Lucien Rouleau, Eileen O’Meara, Anique Ducharme, Jean-Claude Tardif

**Affiliations:** Montreal Heart Institute, Université de Montréal, 5000 Bélanger Est, Montreal, Quebec, Canada H1T 1C8; Montreal Health Innovations Coordinating Center, Montreal Heart Institute, Université de Montréal, 5000 Bélanger Est, Montreal, Quebec, Canada H1T 1C8; Inogen Inc., 859 Ward Dr #200, Goleta, CA 93111, USA; Inogen Inc., 859 Ward Dr #200, Goleta, CA 93111, USA; Montreal Heart Institute, Université de Montréal, 5000 Bélanger Est, Montreal, Quebec, Canada H1T 1C8; Montreal Heart Institute, Université de Montréal, 5000 Bélanger Est, Montreal, Quebec, Canada H1T 1C8; Montreal Heart Institute, Université de Montréal, 5000 Bélanger Est, Montreal, Quebec, Canada H1T 1C8; Montreal Heart Institute, Université de Montréal, 5000 Bélanger Est, Montreal, Quebec, Canada H1T 1C8; Montreal Heart Institute, Université de Montréal, 5000 Bélanger Est, Montreal, Quebec, Canada H1T 1C8; Montreal Health Innovations Coordinating Center, Montreal Heart Institute, Université de Montréal, 5000 Bélanger Est, Montreal, Quebec, Canada H1T 1C8; Montreal Health Innovations Coordinating Center, Montreal Heart Institute, Université de Montréal, 5000 Bélanger Est, Montreal, Quebec, Canada H1T 1C8; Montreal Heart Institute, Université de Montréal, 5000 Bélanger Est, Montreal, Quebec, Canada H1T 1C8; Montreal Heart Institute, Université de Montréal, 5000 Bélanger Est, Montreal, Quebec, Canada H1T 1C8; Montreal Heart Institute, Université de Montréal, 5000 Bélanger Est, Montreal, Quebec, Canada H1T 1C8; Montreal Heart Institute, Université de Montréal, 5000 Bélanger Est, Montreal, Quebec, Canada H1T 1C8; Montreal Health Innovations Coordinating Center, Montreal Heart Institute, Université de Montréal, 5000 Bélanger Est, Montreal, Quebec, Canada H1T 1C8

**Keywords:** Heart failure, Oxygen, Quality of life, Exercise

## Abstract

**Aims:**

The aims of this study were to describe the short-term effects of oxygen therapy on the physiological response and symptoms during ambulation in patients with chronic heart failure (HF).

**Methods and results:**

In this pilot, cross-over, randomized study, subjects with chronic HF underwent two 6-min walk tests (6MWTs) on the same day. They were randomized to either receive oxygen through a portable oxygen concentrator (POC ON) during the first test and no oxygen (POC OFF) during the second test, or vice versa. Endpoints included (i) peripheral oxygen saturation, (ii) heart rate, and (iii) modified BORG scale. A linear mixed model for repeated measures was used for comparisons. A total of 20 participants were included, aged 70 ± 10 years, with the mean left ventricular ejection fraction 33% ± 10% and N-terminal pro-B-type natriuretic peptide 1115 ± 1625 pg/mL. There was no difference in distance walked with or without oxygen supplementation. Oxygen saturation during 6MWT was higher with POC ON [3 min, SpO_2_ + 3.4%, 95% confidence interval (CI) 1.8–5.0%; 6 min, + 2.8%, 95% CI 2.2–3.3%]. Heart rate recovery tended to be better in patients with POC ON (difference 7.4 b.p.m., 95% CI −2.4 to 17.2). Perceived exertion and fatigue were significantly lower with POC ON during exercise (3 min, −0.7, 95% CI −1.2 to −0.2; 6 min, −0.75, 95% CI −1.1 to −0.4; and 3 min into recovery, −0.5, 95% CI −0.8 to −0.2).

**Conclusion:**

Our results suggest that for a same amount of physical activity, supplemental oxygen can improve peripheral oxygen saturation and breathlessness in symptomatic patients with chronic HF.

## Introduction

Heart failure (HF) currently affects between 1 and 2% of all adults, carrying a large burden of complications including mortality, hospitalization, and impaired quality of life.^1^ Therapeutic advances over the past decades improved survival and reduced HF hospitalizations; however, many patients remain highly symptomatic in their daily activites.^2,3^ Approximately half of patients living with HF report difficulty with activities of daily living, most commonly walking and climbing stairs.^4^ This high symptomatic burden highlights the necessity of therapies specifically aimed to address the lack of stamina and poor functional status in patients with symptomatic HF, potentially such as supplemental oxygen.

Long-term oxygen therapy is standard of care in selected conditions, including chronic lung disease associated with severe resting or exertional hypoxaemia as it improves breathlessness, enhances exercise capacity, and prolongs survival.^5,6^ However, there is insufficient evidence to support oxygen therapy in patients without severe hypoxaemia. In chronic HF, the effects of home oxygen therapy have been poorly investigated. While small studies suggested benefits of continuous oxygen therapy with regard to exercise capacity and quality of life, these results were not replicated.^7–9^ As of today, the use of oxygen therapy in chronic HF is not recommended due to lack of sufficient evidence to supports its efficacy and concerns regarding potential deleterious effects of acute oxygen administration on cardiac haemodynamics.^10–12^

The strategy of using home oxygen therapy on an as-needed basis, such as during exercise or other activities of daily living, has not been studied in HF. Portable oxygen concentrators (POCs) can deliver oxygen through a nasal cannula and are sufficiently lightweight to be carried by patients, representing a suitable means of oxygen delivery for ambulatory use.

The aims of this study were to describe the short-term effects of oxygen therapy on the physiological response and symptoms during ambulation in a population of symptomatic patients with HF. We have therefore conducted a proof-of-concept, cross-over, randomized study of POC use during a 6-min walk test (6MWT).

## Methods

The study was compliant with Health Canada regulations and approved by the local ethics board (#2023-3267). All participants signed a written consent form before recruitment. The Montreal Health Innovations Coordinating Center was responsible for study oversight and monitoring. The data that support the findings of this study are available from the corresponding author upon reasonable request.

### Study population

Stable patients followed at the Montreal Heart Institute HF clinic were prospectively screened for study eligibility. Symptomatic, ambulatory patients with borderline hypoxaemia on peripheral oxygen saturation on fingertip pulse oximetry (SpO_2_) were targeted.

The inclusion criteria were as follows: age ≥18 years, chronic HF diagnosis regardless of left ventricular ejection fraction (LVEF), New York Heart Association (NYHA) class ≥2, SpO_2_ ≤ 96% at rest, willingness and ability to use a POC, and capacity to provide informed consent. The exclusion criteria were as follows: contraindication to the use of POC, current or planned hospitalization for HF, acutely decompensated HF requiring intravenous therapy with diuretics or inotropes, acute coronary syndrome within 3 months prior to study enrolment, inability to walk, pregnancy, or any subject who should be excluded in the opinion of the investigator.

Data were collected at the time of randomization and variables included demographics, medical history including cardiac and pulmonary comorbidities, smoking history, NYHA class, LVEF, N-terminal pro-B-type natriuretic peptide (NTproBNP), and cardiovascular medications.

### Intervention

After recruitment, participants were scheduled for a half-day visit at the research centre to perform the 6MWT. Patients were scheduled to perform study procedures at a different date than their medical visit to avoid fatigue related to multiple appointments.

**Figure 1:**
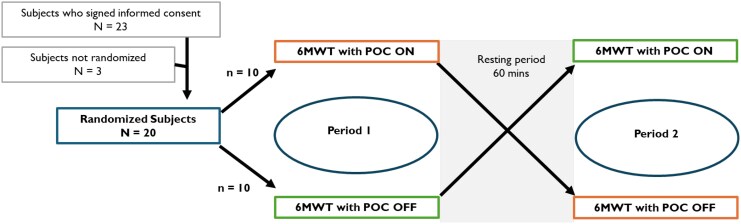
Randomization sequence. Shown is the cross-over randomization design of the study. Subjects were randomized to either perform the first or the second 6-min walk test with the portable oxygen concentrator turned on. The resting period between the two tests was 60 min.

**Figure 2:**
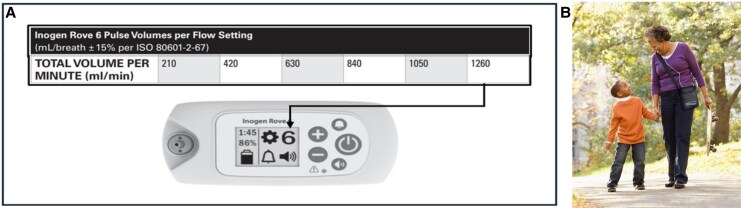
Inogen portable oxygen concentrator. (*A*) Different flow settings of the Inogen portable oxygen concentrator are depicted. In this study, the setting 6 was used, corresponding to oxygen delivery of 1260 mL/min. (*B*) Shown is the Inogen portable oxygen concentrator worn as a shoulder bag.

Each patient performed the 6MWT twice on the same day (*Figure 1*), carrying on both tests the Inogen ROVE 6 POC, as either a shoulder bag or backpack (*Figure 2*). Blinding was not feasible due to the noise generated by the device and the sensation of airflow, making it evident to both patients and investigators whether the therapy was active or inactive.

One 6MWT was performed *with* supplemental oxygen (POC ON) and the other *without* supplemental oxygen (POC OFF). As we anticipated a shorter distance walked on the second 6MWT due to fatigue, patients were randomized in a 1:1 ratio using sealed envelopes to either perform the first test with oxygen followed by the second test without oxygen (POC ON, then POC OFF), or vice versa (POC OFF, then POC ON). There was a resting period of 1 h between the two tests. Regardless of the randomization sequence, patients underwent 15 min with POC ON before each 6MWT to accustom themselves to the sensation of air flow. The device remained ON for the POC ON sequence or was turned OFF 60–90 s prior to exercise for the POC OFF sequence. In order to minimize bias related to carrying additional weight or experiencing discomfort related to the device or cannula, POC was carried and nasal cannula was in place for both sessions.

The ROVE 6 POC weighs 2.2 kg and delivers concentrated oxygen via nasal cannula through pressure swing adsorption process separating O2 molecules from air. It delivers 90% oxygen (±6%/−3%) via pulse dose, providing oxygen only during the inspiratory phase, in contrast to continuous-flow systems that deliver throughout the entire respiratory cycle. For this study, the maximal setting of 6 was used, corresponding to oxygen delivery of 1260 mL/min. The supervising research team ensured that the subject wore the nasal cannula and breathe by the nose through both the sessions of the 6MWT and during the 15 min prior to each session.

### Endpoints

The three endpoints assessed during the 6MWT were the change in (i) peripheral oxygen saturation, (ii) heart rate, and (iii) modified BORG scale, used as a surrogate for the level of difficulty in performing an activity.^13,14^ These endpoints were meant to assess efficacy of oxygen delivery, physiological adaptation to exercise, and perceived exertion. All endpoints were measured before exercise (0 min), during exercise (3 min), at peak exercise (6 min), and at 3 min into recovery (9 min). The oxygen saturation and heart rate were continuously measured through clipped pulse oximeter (Proactive Medical Products Fingertip Pulse Oximeter, model 20110) on the finger, which provided real-time readings. Heart rate recovery was calculated as the difference between heart rate at 6  and 3 min post-exercise.^15^ The modified Borg scale was printed on a large cardboard shown to participants throughout the tests (see Supplementary material online, *Figure S1*). The safety of the study device, Inogen ROVE 6 POC, was monitored and reported for adverse events.

### Statistical analyses

Patient characteristics were reported as means and standard deviations or frequencies and percentages, as appropriate. Normality was tested. The 6MWT distance was reported, and a linear mixed model for repeated measures was used to estimate the difference between POC ON and POC OFF. Specifically, the model accounted for treatment group and sequence of randomization as defined above. From this model, an estimate of the difference between POC ON and POC OFF was obtained and provided with 95% confidence intervals (CIs). A similar approach was used to compare other endpoints between POC ON and POC OFF. The sample size of 20 patients was chosen as a set number given the exploratory nature of this study. Analyses of tolerance to POC were conducted on the intent-to-treat population. Statistical analyses were performed using SAS, version 9.4.

## Results

Twenty-three subjects signed the consent form to participate in the study between October 2023 and November 2023 (*Figure 1*). Among them, three subjects were not randomized: two subjects failed to meet the inclusion criteria and one subject was not selected to continue with the study as the enrolment number of 20 had been reached. A total of 20 patients were randomized, who all completed both sessions of the 6MWT with the POC device turned ON or OFF as assigned.

### Study patients

Participants were aged 70 ± 10 years, comprising 18 males and 2 females with symptomatic HF (*Table 1*). A minority of patients identified as Hispanic (3/20), indigenous (2/20), or Asian (1/20). Aetiology of HF was mostly ischaemic (13/20), and a history of atrial fibrillation or flutter was present in approximately half of patients (9/20). Respiratory disorders included chronic obstructive pulmonary disorder in one patient, asthma in one patient, and obstructive sleep apnoea in six patients. Most subjects had a history of smoking (16/20), three of which were current smokers. The mean LVEF was 33% ± 10%, with a majority (13/20) with LVEF ≤40%, and the mean NTproBNP measured at the screening visit was 1115 ± 1625 pg/mL. The peripheral oxygen saturation of patients was 94.1% ± 2.1% at the screening visit. Guideline-directed medical therapy for HF included beta-blockers in combination with either an angiotensin receptor–neprilysin inhibitor, an angiotensin-converting enzyme inhibitor, or an angiotensin II receptor blocker in all patients. Most patients received a mineralocorticoid receptor antagonist, and a sodium–glucose cotransporter 2 inhibitor. At the clinic visit prior to study completion, two patients had an increase and one patient had a decrease in their oral diuretic dosage.

**Table 1:** Characteristics of the patients at screening

Characteristic	ITT population (*n* = 20)
Age (mean ± SD), years	69.5 ± 10.1
Sex, *n* (%)	
Male	18 (90.0)
Female	2 (10.0)
RACE, *n* (%)	
Asian	1 (5.0)
White	17 (85.0)
American Indian or Alaska Native	2 (10.0)
Cardiopulmonary diseases, *n* (%)	
Coronary artery disease	13 (65.0)
History of angina	14 (70.0)
Coronary artery bypass graft (CABG)	6 (30.0)
Percutaneous coronary intervention (PCI)	10 (50.0)
Peripheral artery disease (PAD)	0 (0.0)
Atrial fibrillation or flutter (paroxysmal)	9 (45.0)
Cardiac resynchronization therapy	5 (25.0)
Implantable cardioverter defibrillator (ICD)	10 (50.0)
Stroke or transient ischaemic attack (TIA)	4 (20.0)
Hypertension	15 (75.0)
Chronic obstructive pulmonary disorder	1 (5.0)
Sleep apnoea	6 (30.0)
Asthma	1 (5.0)
Smoking history, *n* (%)	
Never smoked	4 (20.0)
Currently smokes	3 (15.0)
Formerly smoked	13 (65.0)
NYHA class, *n* (%)	
NYHA Class II	18 (90.0)
NYHA Class III	2 (10.0)
CCS angina class, *n* (%)	
No angina	18 (90.0)
CCS Class II	2 (10.0)
Atrial fibrillation at randomization, *n* (%)	3 (15.0)
LVEF (mean ± SD), %	33 ± 10
NTproBNP (mean ± SD), pg/mL	1115 ± 1625
Medication, *n* (%)	
Beta-blocker	20 (100.0)
Sacubitril–valsartan	18 (90.0)
ACE inhibitor or ARB	2 (10.0)
Mineralocorticoid receptor antagonist	15 (75.0)
Sodium–glucose cotransporter 2 inhibitor	17 (85.0)
Loop diuretic	10 (50.0)

NYHA, New York Heart Association; CCS, Canadian Cardiovascular Society; ACE, angiotensin-converting enzyme; ARB, angiotensin II receptor blocker.

### Six-minute walk test distance

**Figure 3:**
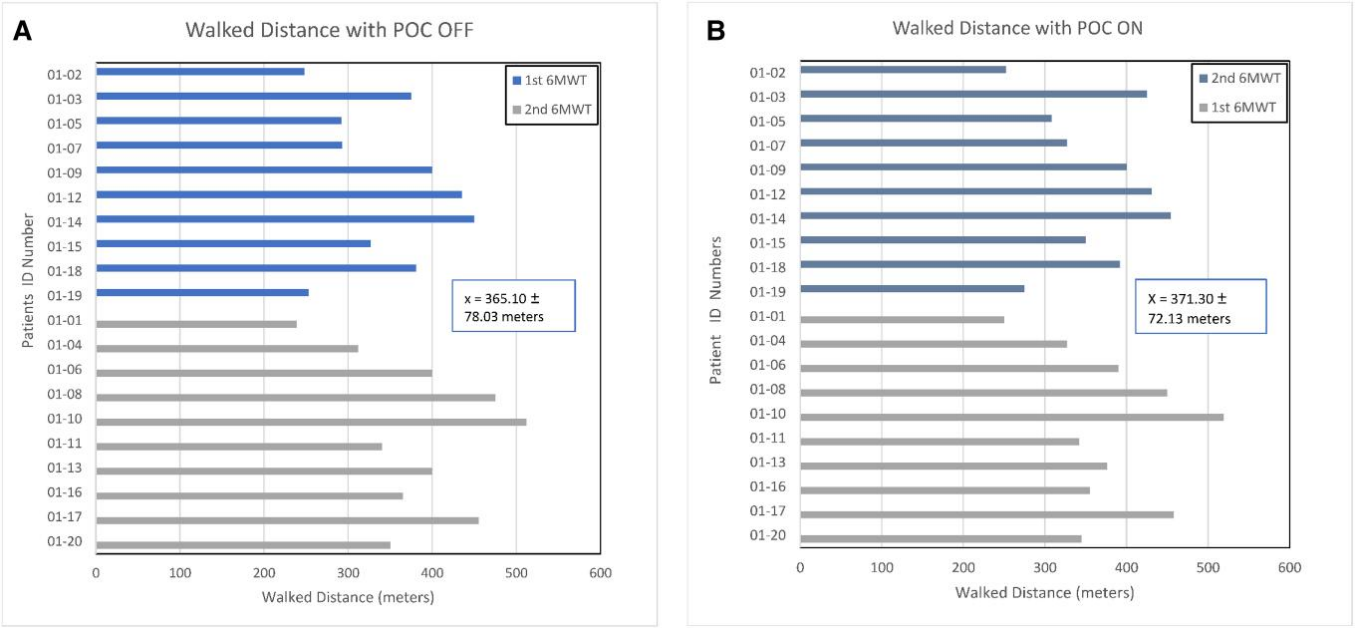
Distance walked during the 6-min walk test. Shown are individual distances walked with POC OFF (*A*) and POC OFF (*B*) for comparison. Individual bars are coloured to inform the sequence of randomization for every subject.

The mean distance walked with POC ON was 371 ± 72 m and POC OFF was 365 ± 78 m (*Figure 3*). The difference in distance walked (+6.2 m with POC ON) was not statistically significant (95% CI −1.0 to 13.4).

### Study outcomes

**Figure 4:**
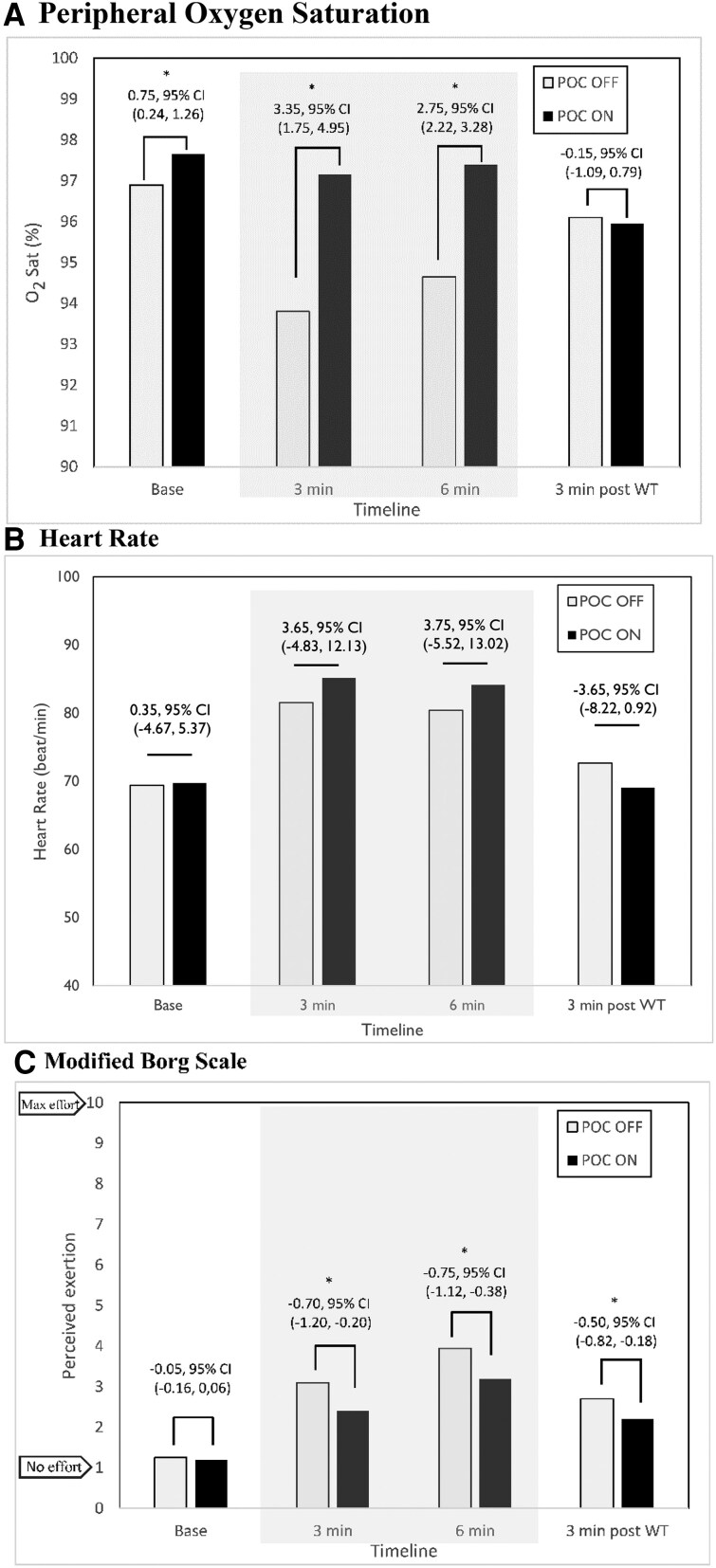
Study outcomes. Shown are mean values of (*A*) peripheral oxygen saturation, (*B*) heart rate, and (*C*) modified BORG scale at baseline, 3 min into exercise, 6 min into exercise, and 3 min into recovery. *Stars identify comparisons with significant difference at the 0.05 alpha level. Grey shaded zones represent values obtained during exercise.

Study outcomes are depicted in *Figure 4*. The oxygen saturation was significantly higher with POC ON compared with POC OFF at baseline (SpO_2_ + 0.8%, 95% CI 0.2–1.3%) and during exercise (3 min, SpO_2_ + 3.4%, 95% CI 1.8–5.0%; 6 min, + 2.8%, 95% CI 2.2–3.3%). The difference in oxygen saturation was no longer observed 3 min after exercise (which represents 3 min after cessation of supplemental oxygen for the POC ON sequence; SpO_2_ −0.2%, 95% CI −1.1 to 0.8%).

The mean heart rate was similar at baseline and during exercise regardless of POC treatment. Three minutes after exercise, there was a trend towards a lower heart rate in the POC ON group (difference −3.7 b.p.m., 95% CI −8.2 to 0.9). The heart rate recovery tended to be better in patients with POC ON compared with POC OFF (15.2 and 7.8 b.p.m., respectively; difference 7.4 b.p.m., 95% CI −2.4 to 17.2).

Perceived exertion and fatigue were similar at baseline and were significantly lower with POC ON during exercise at 3 min (−0.7; 95% CI −1.2 to −0.2) and 6 min (−0.75; 95% CI −1.1 to −0.4). Decreased perception of exertion and fatigue carried on 3 min into recovery, where the Borg scale value was on average −0.5 (95% CI −0.8 to −0.2) points lower when the 6MWT was performed with POC ON. No patient reported anginal symptoms during exertion.

Two patients developed hypoxia (SpO_2_ < 92%) during exercise. The first reached an SpO_2_ of 82% at 3 min with POC OFF; the second reached an SpO_2_ of 91% at 6 min with POC OFF. In the first patient, the Borg score improved by one point with oxygen (Borg 5 with POC ON vs. Borg 6 with POC OFF at 6 min). In all other comparisons for these two patients, Borg scores were identical.

### Safety

One participant reported a modest distortion in smell during the study intervention with POC turned ON. This event was classified as mild and plausibly related to the use of the Inogen ROVE 6 POC. There were no serious advents reported in the study.

## Discussion

This study was designed to assess whether supplemental oxygen impacts the physiological response to exercise and symptoms during ambulation in HF patients. In this randomized, proof-of-concept study involving symptomatic patients with chronic HF, repeated 6MWTs were performed and resulted in a similar distance walked with or without oxygen delivered through a POC. However, supplemental oxygen improved selected exercise parameters, specifically peripheral oxygen saturation, heart rate recovery, and perceived exertion or fatigue.

There were few previous studies evaluating the use of home oxygen in HF, yielding mixed results. In a study comparing long-term oxygen therapy for at least 15 h per day to usual treatment, there was no impact on quality of life.^8^ A more recent analysis assessed nocturnal oxygen for 8 h per night for 1 month in HF patients, reporting a 17% increase in 6MWT distance and improved quality of life.^9^ However, the latter study lacked a control group, limiting the establishment of a cause–effect between oxygen therapy and improvement.

Our study differs from previous work by employing a selective approach to oxygen use, administering it only during exercise when cardiac demand is higher. Since HF is associated with a blunted increase in cardiac output during exercise, it could be hypothesized that supplemental oxygen helps meet higher metabolic needs.^16^ The acute lack of oxygen during exercise is supported by a recent study showing that 25% of patients with HF and preserved ejection fraction develop low-grade hypoxaemia during cardiopulmonary exercise testing, despite the absence of pulmonary disease.^17^ In our study, where a lighter exercise regimen was performed, we observed hypoxaemia in only two patients, but did detect improvement in physiological parameters and symptoms with oxygen.

The 6MWT was chosen as it is self-paced and representative of the activities of daily living in the target subject population. The test is commonly performed to measure treatment response in subjects with HF or respiratory conditions.^18–20^ All patients in the study were able to complete the 6MWT, therefore successful in providing a proxy for daily exercise.^21^ While 6MWT distance was recorded, our primary focus was on assessing changes during a fixed amount of exertion, and therefore not included as a study endpoint. Although the distance walked was numerically higher with oxygen, the difference was not clinically meaningful.

The oxygen saturation during exercise was higher with POC ON, reflecting enhanced systemic oxygen delivery. Accordingly, there was a tendency for improvement of heart rate recovery with POC ON. Prior studies indicate that exertional desaturation in HF patients without underlying lung disease is associated with adverse haemodynamics and increased physiologic shunting.^17^ Interventions that improve oxygenation, such as portable oxygen delivery, may help mitigate haemodynamic stress and support autonomic recovery post-exercise. By blunting sympathetic activation and promoting parasympathetic reactivation, oxygen may contribute to improved heart rate recovery. While speculative, this is consistent with prior studies showing improved exercise responses with acute oxygen supplementation.^22–24^ Heart rate recovery has also been correlated with peak oxygen consumption^25^ and improved cardiovascular outcomes in HF patients.^26^

The highest mean perceived Borg scale was 4.0 at 6 min in the POC OFF group, representing ‘a little out of breath’, while at the same timepoint POC ON achieved 3.2, representing ‘comfort zone, everything is fine’, with a between-group difference of 0.75 points. However, these results must be interpreted with caution, as prior work has suggested minimally important clinical differences for the Borg scale of 0.9 in pulmonary hypertension and 1.0 in chronic lung disease.^27,28^ Patients with HF report that symptom control and functional improvement are part of their top priorities.^29^ Therapeutic advances have led to improved survival in HF patients, but most clinicians agree that for some patients, symptom control may take precedence over longevity.^30^ In addition, although speculative, if a person knows that a given activity will provoke breathlessness, they might be avoiding that activity in the future.^31^ By removing this mental barrier through the use of supplemental oxygen, patients might be more inclined to engage with the activities of daily living. To address these important questions, future studies are warranted to assess treatment avenues aimed at improving symptoms, and potentially functional status, including oxygen delivery.

The trade-off between the burden of oxygen delivery equipment and improvements in perceived well-being during exercise is an important consideration, as the practical challenges of using POCs can lead to anxiety about mobility and dependency.^32,33^ While oxygen therapy may enhance quality of life in HF patients, these burdens must be considered alongside the benefits, offering valuable insights for optimizing oxygen use in HF patients.

Specifically, additional studies should focus on the home setting and incorporate outcome measures such as quality-of-life questionnaires, ambulation trackers, such as pedometers, and clinical events as exploratory outcomes. Importantly, these further studies should also monitor potential safety concerns associated with home oxygen therapy. Previous reports have indicated detrimental effects of hyperoxia, particularly in the acute setting, such as short-term deterioration of haemodynamic parameters.^10,11,34^ The absence of serious adverse events in our study is a preliminary indication of the innocuity of the intervention in HF patients.

### Limitations and strengths

Although this pilot study enrolled a small number of patients, its randomized design minimized the introduction of bias. The randomized cross-over nature of the study helped account for fatigue associated with both tests. However, due to the non-blinded nature of the study, oxygen flow may have influenced patients’ perception of exertion, as airflow has previously been shown to alleviate breathlessness, potentially through the placebo effect.^35^ Nevertheless, airflow is unlikely to have affected objective parameters such as oxygen saturation or heart rate. Additionally, while having patients carry equipment during both tests helped maintain comparable groups, it imposed additional constraints on the POC OFF group, which may have hindered their performance. While we acknowledge that the use of patient-reported outcomes in non-blinded studies presents methodological limitations, these measures retain clinical relevance, particularly in studies targeting symptomatic relief, when interpreted within the appropriate context and with due consideration of potential sources of bias. Although smoking history was common, chronic obstructive pulmonary disease was formally diagnosed in only one patient (LVEF 30%, NTproBNP 344 pg/mL), and available pulmonary function tests in others did not support significant obstructive lung disease as the primary cause of exertional dyspnoea (see Supplementary material, Supplementary material online, *Table S1*). Limitations also included the absence of respiratory rate monitoring and electrocardiographic assessment during exertion.

## Conclusions

This exploratory study evaluated the efficacy and safety of delivering supplemental oxygen therapy to subjects with stable chronic HF during the 6MWT. Our results demonstrated the ability of supplemental oxygen to improve oxygen saturation, heart rate recovery, and breathlessness, for the same amount of physical activity. Larger studies are necessary to confirm these findings and applicability to a broader population.

## Supplementary Material

oeaf074_Supplementary_Data

## Data Availability

The data are available upon reasonable request to the corresponding author.

## Lead author biography

Dr Maxime Tremblay-Gravel, MD, MSc, is a cardiologist and clinician-scientist at the Montreal Heart Institute and an assistant clinical professor at the Université de Montréal. He completed his adult cardiology training at the Université de Montréal, followed by fellowships in cardiovascular genetics, advanced HF, and echocardiography at Stanford University. His clinical and research interests focus on genetic cardiomyopathies, including hypertrophic and dilated forms, as well as cardiac transplantation and HF management.
