# Supplementary material for: Oxygen supplementation in ambulatory patients with heart failure: a randomized proof-of-concept study
Source: Eur Heart J Open. 2025 Jun 11;5(3):oeaf074. doi: 10.1093/ehjopen/oeaf074 (PMC12205361; doi:10.1093/ehjopen/oeaf074)
Supplement: oeaf074_Supplementary_Data [file oeaf074_supplementary_data.pdf]

## Supplemental material online

**Figure S1. Modified BORG scale**

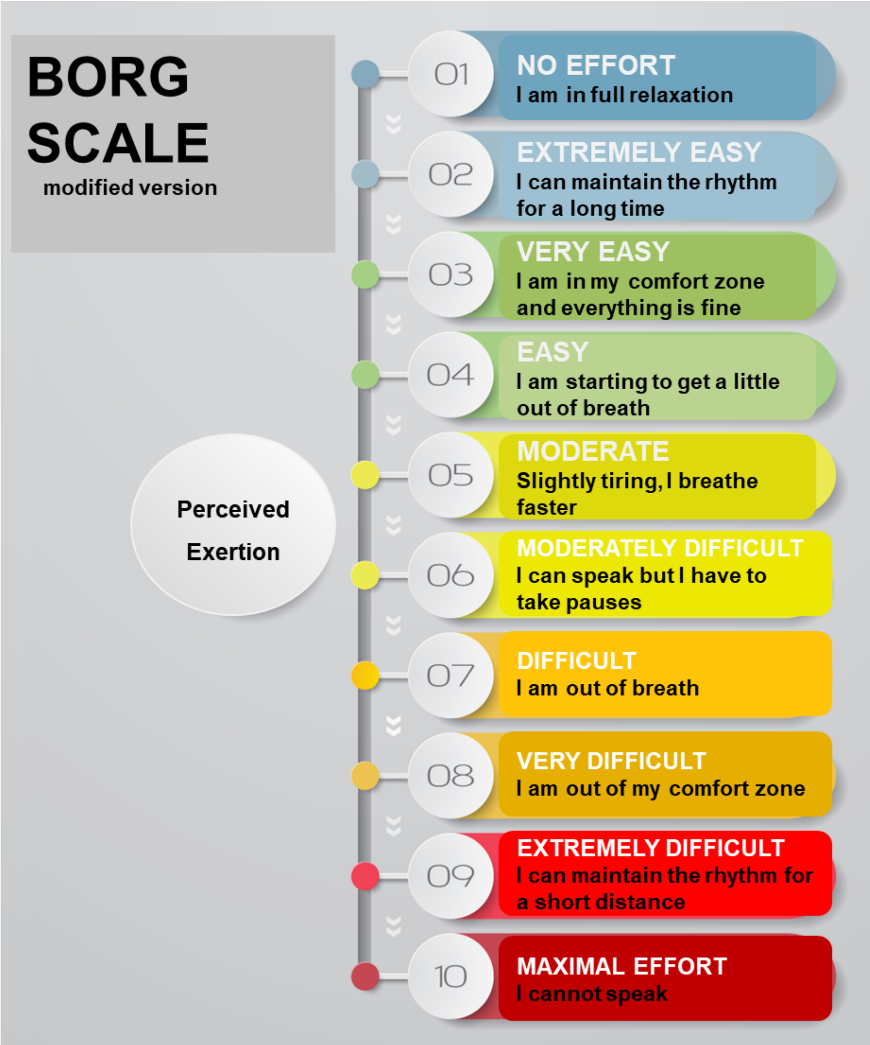

The mBorg scale is a numerical intensity rating that ranges from 1 to 10. Each rating represents the level of difficulty in performing an activity. An intensity rating of 1 denotes the subject does not require any effort to perform an activity, while a score of 10 denotes that the subject requires maximum effort to speak while performing an activity.

**Table S1. Pulmonary function tests**

| Patient ID | Measured FEV1 (L) | % Predicted FEV1 | FEV1/FVC | % Predicted corrected DLCO |
|---|---|---|---|---|
| **01-06** | 3.7 | 95 | 79 | 60.9 |
| **01-20** | 1.9 | 87 | 78 | 83.6 |
| **01-21** | 3.1 | 106 | 81 | N/A |

FEV1 denotes forced expiratory volume in 1 second; FVC, forced vital capacity; DLCO, diffusing capacity of the lung for carbon monoxide.
